# Copper(II) ions affect the gating dynamics of the 20S proteasome: a molecular and *in cell* study

**DOI:** 10.1038/srep33444

**Published:** 2016-09-16

**Authors:** Anna Maria Santoro, Irene Monaco, Francesco Attanasio, Valeria Lanza, Giuseppe Pappalardo, Marianna Flora Tomasello, Alessandra Cunsolo, Enrico Rizzarelli, Ada De Luigi, Mario Salmona, Danilo Milardi

**Affiliations:** 1 Istituto di Biostrutture e Bioimmagini - CNR Sede di Catania, Via P. Gaifami, 9- 95126 Catania, Italy; 2 Fondazione RiMed, Via Bandiera 11, 90133, Palermo, Italy; 3 Dipartimento di Scienze Chimiche, Università di Catania, Viale Andrea Doria 6, 95125 Catania, Italy; 4 IRCCS-Istituto di Ricerche Farmacologiche “Mario Negri”, Via Giuseppe La Masa 19, 20156, Milano, Italy

## Abstract

Due to their altered metabolism cancer cells are more sensitive to proteasome inhibition or changes of copper levels than normal cells. Thus, the development of copper complexes endowed with proteasome inhibition features has emerged as a promising anticancer strategy. However, limited information is available about the exact mechanism by which copper inhibits proteasome. Here we show that Cu(II) ions simultaneously inhibit the three peptidase activities of isolated 20S proteasomes with potencies (IC_50_) in the micromolar range. Cu(II) ions, in cell-free conditions, neither catalyze red-ox reactions nor disrupt the assembly of the 20S proteasome but, rather, promote conformational changes associated to impaired channel gating. Notably, HeLa cells grown in a Cu(II)-supplemented medium exhibit decreased proteasome activity. This effect, however, was attenuated in the presence of an antioxidant. Our results suggest that if, on one hand, Cu(II)-inhibited 20S activities may be associated to conformational changes that favor the closed state of the core particle, on the other hand the complex effect induced by Cu(II) ions in cancer cells is the result of several concurring events including ROS-mediated proteasome flooding, and disassembly of the 26S proteasome into its 20S and 19S components.

The 26S proteasome is a multicatalytic enzyme that can be disassembled into two subcomplexes: the 20S proteasome, also known as the core particle (CP) and a regulatory 19S particle (RP) that may bind either to one or both sides of the CP^1^. The CP is a 700 kDa multisubunit assembly composed by 7 different α- and 7 different β-subunits arranged as a barrel-shape pile of four heptameric rings. The two outward α-rings keep hold of two inner β-rings containing the proteolitically active β-subunits, thus acquiring a final α7/β7/β7/α7 structure. In the catalytic chamber of the CP there are two replicas of three different proteolytic β subunits, i.e. β1, β2 and β5, which exhibit caspase-like (C-L), trypsin-like (T-L) and chymotrypsin-like (ChT-L) activity, respectively^2^,^3^,^4^. Notably, substrates reach the catalytic sites only through a gated channel in the α–ring^5^,^6^,^7^. The 20S particle is significantly present in live cells where it contributes to protein homeostasis by degrading oxidized and misfolded protein substrates^8^. Proteasomes, due to their enhanced activity in malignant cells^9^, have emerged over the past decade as very promising targets in the treatment of a number of tumors with the competitive proteasome inhibitors (PIs) Bortezomib and Carfilzomib approved as ground-breaking therapies for multiple myeloma^10^,^11^. However, despite the initial success of these treatments, clinicians using competitive PIs had to face soon problems of relapse, drug resistance and severe side effects^12^. All these hurdles have prompted studies focusing on alternative strategies aimed at interfering either with proteasome activity through allosteric effects and/or with other oncogenic pathways^13^,^14^,^15^. In this context, copper complexes have been considered promising anticancer drugs^16^,^17^,^18^,^19^,^20^. Indeed, due to their altered metabolism, cancer cells have a different response than normal cells to copper overload^21^ and high serum copper concentrations (ranging from 10 to 30 μmol/L) are associated with a variety of cancers including lymphoma, sarcoma, carcinomas, cervical, breast, stomach and lung cancers^22^,^23^,^24^,^25^. Many anticancer copper complexes with ionophores (e.g 8-hydroxyquinoline, 8-OHQ, clioquinol, CQ, and dithiocarbamate, DTC) show a significant proteasome inhibitory capacity^17^,^26^. Of note, all those reports point to a key role played by Cu(II) ions in promoting proteasome inhibition but incomplete information is available about the molecular mechanisms underlying these biological evidences.

To address these issues, here we investigate the ability of Cu(II) ions to inhibit the peptidase activities, assembly and gating mechanisms of the 20S proteasome in cell-free conditions. Finally, we validate the significance of our results by investigating the Cu(II)-mediated proteasome inhibition in live Hela Cells.

## Results and Discussion

### Cu(II) ions inhibit 20S proteasome activities without altering the CP assembly

**Figure 1:**
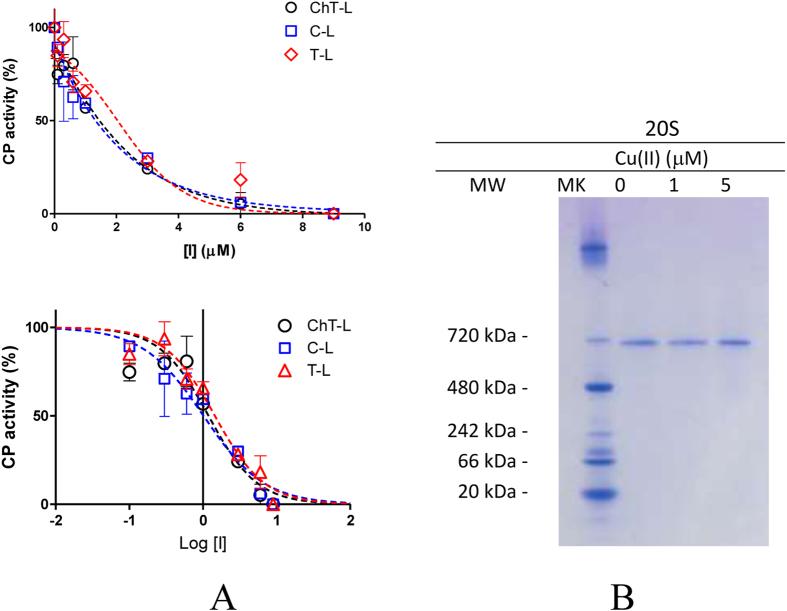
(**A**) Normalized concentration−response plot for Copper(II)-mediated inhibition of ChT-L, T-L and C-L residual activities of hCP (upper panel). The lower panel report the peptidase activities as a semilog plot fitted by Equation 1. IC_50_ values for the distinct peptidase activities and the related fitting parameters are reported in Table 1. (B) Native gel electrophoresis of free 20S proteasome (0.2 μg). hCP was exposed to increasing amounts of Cu(II) ions for 60 min and silver stained as described in the experimental section.

First, we used fluorogenic substrates to evaluate the effect of Cu(II) ions on the distinct peptidase activities of human 20S proteasome preparations (hCP). Cu(II) ions inhibited all proteolytic activities with similar potencies in the micromolar range (see Fig. 1A and Table 1) consistent with previous reports^27^.

**Figure 2:**
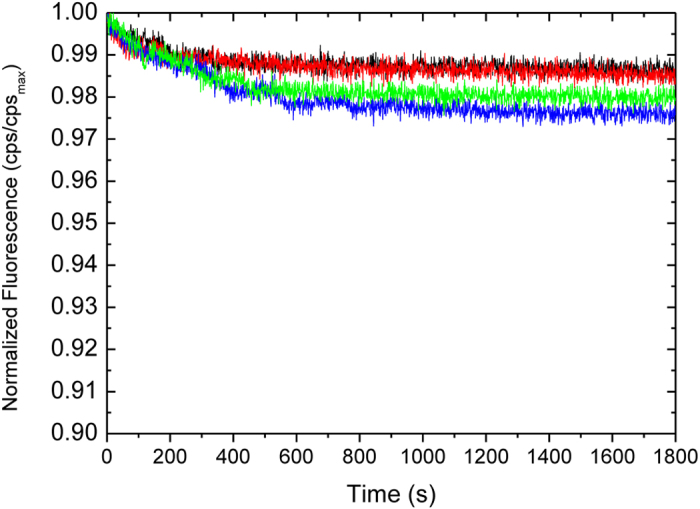
Copper(II)-catalyzed ROS formation monitored in 25 mM Tris buffer, pH 7.6 by fluorescence intensity decrease as a function of time for: 5 μM Cu(II) (red line); hCP 2 nM, 5 μM Cu(II) and 29 μM DTT (black curve); 29 μM DTT (green curve); 5 μM Cu(II) and 29 μM DTT (blue curve). Due to the presence of 1 mM DTT in all commercial stock solutions of hCP, control experiments at 29 μM DTT were performed to mimick the effect of Cu(II)/DTT in a hCP-free solution.

Currently, there are only few molecules known to inhibit the ChT-L, C-L, and T-L proteasome activities to the same extent^28^. In principle, these agents are thought to inactivate the CP by upstream events involving protein damage (e.g. oxidation, disassembling of the enzyme into α and β subunits)^29^,^30^ or, as recently reported, to allosteric effects involving proteasome gating^31^. To address these issues, we performed native gel electrophoresis of purified hCP after 1 h incubation with 1 and 5 μM Cu(II) ions (Fig. 1B). In all cases, only a single band is present at 700 kDa. Thus, we can exclude that Cu(II)-treated hCP could disassemble in α and β subunits. Reduction of Copper(II) to Copper(I) via Fenton chemistry is known to catalyze the production of ROS in the presence of O_2_ and reducing agents such as, for example, ascorbate or DTT. Therefore, due to the presence of DTT in all 20S samples, it is important to investigate the presence of Cu(I) and ROS in the solutions employed for proteasome assays. To this aim we i) assayed the presence of Cu(I) species in Cu(II)-loaded hCP samples by UV spectroscopy using bathocuproine disulfonic acid sodium salt (BCS) (see Figure S1 of Supporting Information) and ii) monitored the ROS production using fluorescein as a probe whose fluorescence intensity decreases upon reacting with ROS^32^. Fluorescence data reported in Fig. 2 evidenced that copper-treated samples were not able to catalyze production of ROS. Therefore, we can conclude that, in the experimental conditions adopted in proteasome assays, Cu(II) is not reduced to Cu(I). Proteasome inhibition may be thus due to a direct binding of Cu(II) ions to the protein. In accordance to this hypothesis, the lower inhibition potency observed for Zn(II) - a red-ox inactive metal ion - with IC_50_ values ranging from 4.16 to 5.99 μM (see Table 1) reflect the relative stabilities of metal complexes described in the Irving-Williams series.

### Cu(II) ions affect CP gating

Incubation of yCP crystals with millimolar concentrations of Cu(II) ions resulted in their rapid degradation thus making impossible any X-ray analysis of the Cu(II)-20S complex (M. Groll personal communication). Therefore, to gain insights into the possible Cu(II)-induced conformational changes, we employed TrP fluorescence analysis of the isolated 20S hCP^33^.

**Figure 3:**
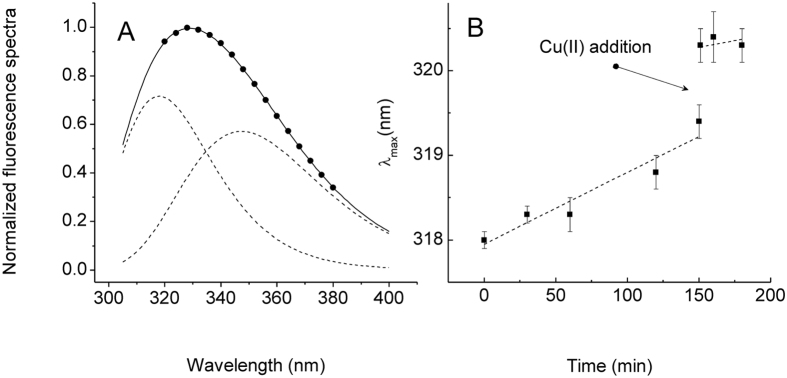
(**A**) Typical deconvolution of normalized tryptophan fluorescence spectra of the 20S proteasome at pH 7.6 recorded immediately after the addition of 0.018% SDS (normalized experimental spectra – black points; fitting curve - solid line; spectral components – dotted lines). (**B**) Plot of the wavelength shift of the first spectral component *vs* time (closed squares). The two dotted lines represent the linear fits of the values obtained before (t < 150 min) and after (t > 150 min) the addition of Cu(II) ions.

In particular, fluorescence spectra of the isolated 20S proteasome may be deconvoluted into two components. The first of these bands is ascribable to Trp13(α6) residues which are located at the entrance of the channel^33^. Therefore, a red-shift of the first component may be associated to a gate opening of the 20S proteasome. Here, we exploited this effect to investigate the role of Cu(II) ions in CP gate closing. In particular, the fluorescence emission spectra recorded over time show that SDS induces a slight time-dependent decrease of the total intensity of the fluorescence spectrum in agreement with previously reported data^33^. Next, we recorded the fluorescence spectra obtained immediately (t = 0 min) and after 30 min the addition of 100 μM Cu(II) ions to the SDS-activated CP and deconvoluted the fluorescence spectra into the contributions from each type of tryptophan as described in the experimental section (see Fig. 3 panel A). We found that the first component underwent spectral red shifts of max 2 nm if monitored over time. This red shift likely results from increased solvent access of the Trp residue located at the channel entrance upon SDS-induced gate opening. The addition of a large excess of Cu(II) ions (100 μM) to the SDS-activated CP reduced this effect as evidenced by the decreased rate of red-shift (Fig. 3 panel B). Next, in order to properly compare TrP fluorescence data with proteasome activity we also repeated peptidase assays in the presence of SDS (see Figure S2 and Table S1. All the IC_50_ values did not result dependent on the presence of SDS thus strengthening the significance of our comparison with the steady state fluorescence experiments. This evidence is consistent with an effect of Cu(II) ions on the CP gating mechanisms which result shifted toward the closed conformation.

### Cu(II) ions do not inhibit the α3ΔN mutant yeast proteasome

**Figure 4:**
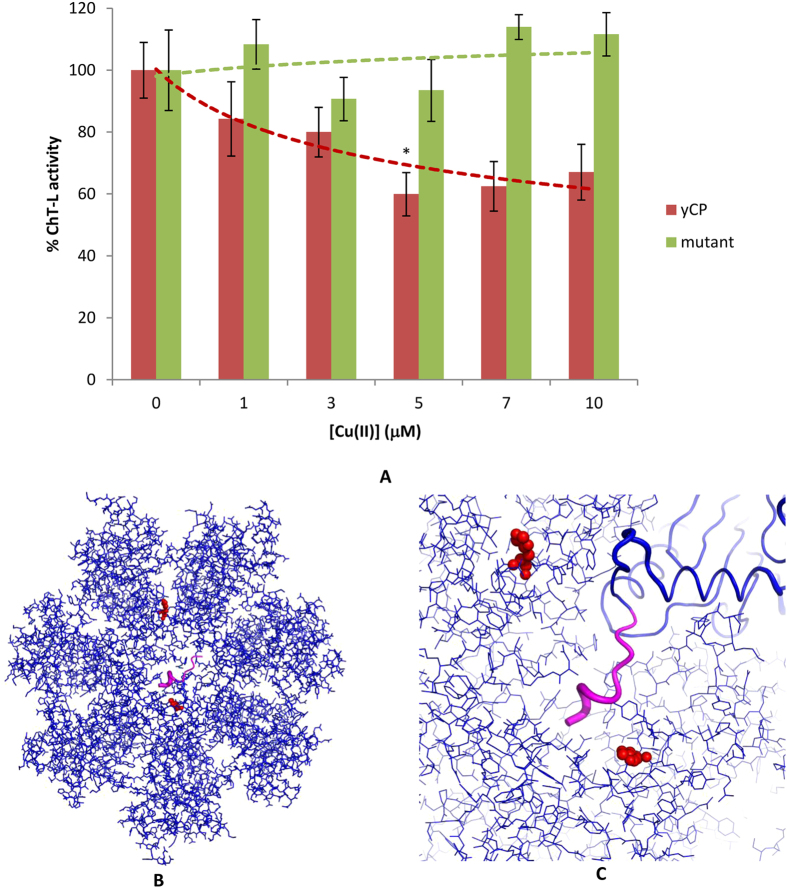
(**A**) Copper(II)-inhibited ChT-L peptidase activity of yCP (red bars) and the α3ΔN mutant (green bars); (pdb code: 4Q1S). By one-way ANOVA and Turkey’s multiple comparison test, ChT-L activity in the presence of Cu(II) 5 μM are found to be significantly lower (*p < 0.05) than those at 3 μM. (**B**) Top views of the α-rings of yCP. C); Magnified picture of the yCP gate. The first nine residues of the α3 subunit, which are not present in the α3ΔN mutant, are represented in magenta. Two histidines (α6-H14 and α1-H6) located at the entrance of the gate and near the N-terminal segment of the α3 subunit are evidenced in red. A third His residue (α3-H30) is far away from the gate and is not shown.

All the experimental data collected so far reconcile with the hypothesis that Cu(II) ions have a direct influence on the gating mechanisms of the 20S proteasome. To further validate this hypothesis, we assayed the effect of Cu(II) ions on the ChT-L peptidase activity of a mutant proteasome (α3ΔN) where deletion of the first nine N terminal residues of the α3 subunit results in permanently open gate conformation^6^. Consistent with previous results, Cu(II) ions did not affect proteasome activity (Fig. 4, panel A). This result confirms the effect of Cu(II) ions in modulating equilibrium between the open and closed gate. It is worthy to remark here that several histidine residues are located at the entrance of the channel and, more specifically in the proximity of the N-terminal tail of the α3 subunit (Fig. 4 panels B and C). It is tempting to speculate that these His residues may play a major role in driving the Copper(II) –mediated gating dynamics.

### Cu(II)-induced proteasome inhibition in HeLa cells

**Figure 5:**
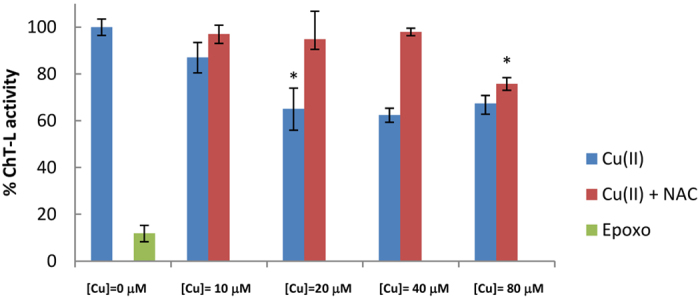
Proteasome inhibition effects of Cu(II) ions monitored by luminescent probes (ProteasomeGlo^©^) in HeLa Cells. Cells were treated with different amounts of Cu(II) (blue bars) and also in the presence of (100 μM) NAC (red bars) for 24 h. The effect of epoxomycin (2 nM) on proteasome activity is also reported as a control (green bar). Data from three independent experiments were normalized to untreated controls and are reported as means ± standard deviation. By one-way ANOVA and Turkey’s multiple comparison test, ChT-L activity in the presence of Cu(II) 20 μM are found to be significantly lower (*p < 0.05) than those at 10 μM Cu(II). Anlogously, ChT-L activity in the presence of Cu(II) 80 μM are found to be significantly lower (*p < 0.05) than those at 40 μM Cu(II) if NAC is present in the medium.

We have shown that, in cell-free conditions, Cu(II) ions may directly bind the 20S proteasome and influence its gating dynamics. However, it is still unclear if this inhibition mechanism may also occur in live cells. To address this question we determined proteasome ChT-L activity in HeLa cells incubated for 24 h with different amounts of Cu(II) ions (ranging from 10 to 80 μM) (see Fig. 5 panel A, blue bars). In proteasome activity assays performed with live cells one should remind that copper, in order to reach cytosolic proteasomes, must cross the cell membrane. In doing this, the metal ions are managed by a complex copper-chaperone machinery and may competitively bind to a large number of copper-binding proteins. Therefore it is quite conceivable that, when added to the cell medium, copper must be present at higher concentrations than tube tests in order to exert its inhibitory effect. Furthermore, serum copper levels are increased in various types of cancers ([Cu] ~ 10–30 μM)^22^,^23^,^24^,^25^. Likely these levels may be even increased in the proximity of affected tissues. Based on these evidences we measure proteasome activities in the presence of Cu(II) ions ranging from 10 to 80 μM. HeLa cells were chosen as a representative model of cancer cells because their growth is not inhibited in Cu(II)-supplemented media up to 100 μM thus allowing an unbiased analysis of proteasomal activity^34^,^35^.

We observed that the ChT-L activity decreases down to 60% when Cu(II) ions are added to the medium at concentrations ranging from 20 to 80 μM. This trend does not display a classical dose-response profile: in particular the ChT-L activity measured at 80 μM Cu^2+^ is slightly higher than samples incubated at lower Cu^2+^ concentrations. Notably, this trend was also reproduced using the peptide TED, an intracellular proteasome reporter developed by some of us (see Figure S3 of Supporting information)^36^. In an attempt to rationalize this behavior, we first measured the cellular uptake of metal ions by flow cytometry using Phen Green, a fluorescent probe that decreases its fluorescent signal in the presence of metal ions^37^. Cells loaded with Phen Green under control conditions exhibited a fluorescent signal which was here considered as the reference maximal intensity signal (representative flow cytometry 1D and 2D plots are reported in Figure S4 of Supporting Information). Exposure of cells to exogenous Copper(II) entrained the drop of Phen Green fluorescent intensity in most of the cell population considered. This decrease is dose-dependent, although at 40 μM Cu^2+^ reaches a plateau, in agreement with previous reports^38^.

Cu^2+^ promote pro-apoptotic pathways by oxidative stress^39^. Therefore, in order to verify if proteasome inhibition results from a Copper(II)-mediated redox phenomenon, HeLa cells were pretreated for 24 h with 100 μM of NAC (N-acetyl cysteine), thus increasing the antioxidant defense of the cell (see Fig. 5, red bars)^40^. The results evidenced that, in the range 20–40 μM, copper-induced proteasome inhibition was completely abolished in cells pretreated with NAC. On the other hand, NAC treatment did not overcome proteasome inhibition when the concentration of exogenous copper was 80 μM. It is worthy to remind here that, although intracellular copper load reaches its maximum when the cells are grown in a 40 μM medium, excess extracellular Cu(II) ions may still exert their oxidant effects. These evidences indicate that proteasome inhibition by copper may be attributed only in part to red-ox effects, and that other phenomena, likely involving a direct binding of the metal ions to the proteasome should be invoked.

### Proteasome disassembly in Cu(II)-loaded Hela Cells

**Figure 6:**
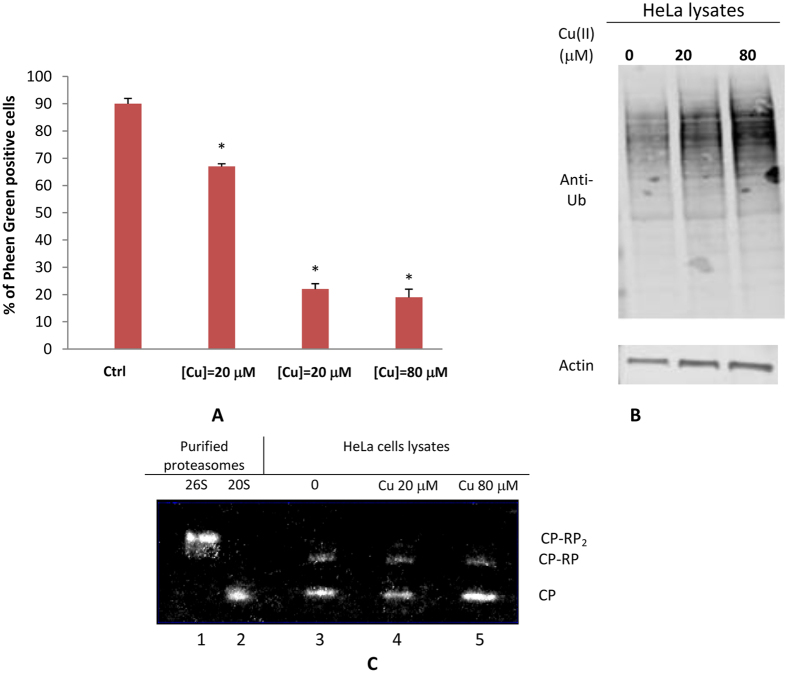
(**A**) Percentage of cells with fluorescence values included in the yellow area (or in the gate M1) in the representative flow cytometry 2D and 1D plots reported in the Supplementary Information. Significant difference from control value was indicated by *(p < 0.05) (one-way ANOVA with Tukey’s post hoc test) (**B**) Cu(II) enhanced the accumulation of ubiquitinated proteins in Hela cells. Hela cells were treated with 20 and 80 μM of Cu(II) for 24 hours, and then ubiquitinated proteins were detected by Western blot (**C**). Proteasome disassembly following HeLa cells incubation in the absence (lane 3) and with 20 (lane 4) and 80 μM Copper(II) (lane 5). Isolated 20S and 26S proteasomes are also analyzed as a control (lanes 1 and 2). At 24 h of incubation equal numbers of cells were harvested, and lysates were separated by nondenaturing PAGE and proteasome activity was evidenced by in-gel peptidase activity. The proteasome species are indicated as CP-RP_2_, doubly capped 26S; CP-RP, singly capped 26S; and CP, uncapped 20S.

Previous reports have evidenced an increased pool of uncapped 20S in cells grown in oxidative stress conditions^41^. In fact, differently from the holoenzyme 26S, whose activity is strictly dependent on the upstream ubiquitination machinery, the 20S is less vulnerable to oxidative stress and may thus face more rapidly and effectively the increasing burden of oxidized and misfolded proteins in a ubiquitin-independent manner. Notably, the effect of exogenous copper overload on the ubiquitin-dependent degradative pathway exhibited a roughly linear dose-response relationship as evidenced by the accumulation of polyubiquitinated proteins reported in Fig. 6B. Therefore, we evaluated the effect of Cu(II) ions on the 26S/20S ratio by visualizing proteasome bands in non-denaturing gels of Cu(II)-treated Hela cells lysates (see Fig. 6C). It is evidenced that copper treatment alters the 20S/26S ratio. In particular, although at 20 μM the intensity of the bands relative to CP, CP-RP and CP-RP_2_ does not vary with respect to control, by contrast at 80 μM the amount of CP increases and a concomitant reduction of CP-RP_1_ and CP-RP_2_ bands is observed. These data parallel the trend of ChT-L activity (see Fig. 5A) and suggest that if, on one hand, Cu(II) inhibits proteasome peptidase activities, on the other hand it may, trough oxidative stress, promote the disassembly the 26S proteasomes with a consequent increase of the amount of free 20S.

## Conclusions

Data reported here demonstrate that, in cell-free conditions, Cu(II) ions affect the gating dynamics of the 20S proteasome by promoting an irreversible closure of the α-rings. This effect does not depend on the red-ox activity of Cu(II) ions since it may be also observed, albeit to a lower extent, when the CP is incubated with red-ox inactive Zn(II) ions. However, ROS are present in HeLa cells grown in Cu(II)-supplemented media and concur to proteasome inactivation. Moreover, although ubiquitin-dependent proteolysis is inhibited by copper in a dose dependent manner - as evidenced by the accumulation of polyubiquitinated proteins - the response of proteasome peptidase activity to elevated Copper(II) levels measured in live HeLa cells is not linear. In fact, a slight recovery of proteasome activity was observed for HeLa cells cultured in 80 μM Cu(II)-supplemented medium with respect to samples grown in 40 μM Cu(II). Notably, non-denaturing gels of cell extracts from Cu(II)-treated samples have shown that the 20S/26S ratio is increased in the presence of increasing copper amounts. Therefore, the effect of Cu(II) on gating dynamics observed in tube tests might coexist, in live cells, with other phenomena which include oxidative stress (as evidenced by the rescuing effect of NAC) and disassembly of the 26S into its 20S and 19S subparticles. These two last events result to be closely intertwined and occur at higher Cu(II) concentration. However, an increment of the relative amount of 20S may counterbalance proteasome inhibition thus contributing to the overall apparent effect of Cu(II) on proteasome inhibition. Conclusively, Cu(II) may affect proteasome activity at multiple levels either acting as a gatekeeper or promoting oxidative phenomena which may compromise the assembly of the holoenzyme. A clear knowledge of all these phenomena is expected to better guide the design of novel and more effective Cu(II)-based PIs.

## Methods

### Chemicals

Purified human 20S proteasome (hCP) and the fluorogenic substrates Suc-LLVY-AMC, Z-Leu-Leu-Glu-AMC, and Ac-Arg-Leu-Arg-AMC used to assay the ChT-L, T-L, and C-L activity, were from Boston Biochem (Cambridge, MA, USA). Bathocuproinedisulfonic acid disodium salt (BCS), dithiothreitol (DTT), N-Acetyl-L-Cysteine (NAC), Sodium dodecylsulphate (SDS) as well as CuCl_2_ and Zn(NO_3_)_2_ were from Sigma-Aldrich (St. Louis, MO). Proteasome inhibitors Epoxomicin were obtained from Enzo Life Science. Wild type 20S proteasome isolated from *Saccharomices Cerevisiae* (yCP) and a mutant (α3ΔN) where nine residues of the N-terminal tail of the α3 subunit are deleted to provide a permanently open-gate conformations were isolated as described elsewhere^42^.

### Peptidase activities of isolated hCP

Peptidase activities of hCP (2 nM) were assayed on in 50 μL Tris 25 mM pH 7.6, at 37 °C with 100 μM of the fluorogenic substrate. hCP was incubated with Cu(II)/Zn(II) ions for 1 h prior activity assays. The fluorescence signal activated by peptide cleavage was recorded at 440 nm (excitation at 360 nm) for 45 min, by a Varioskan (Thermo^©^) plate reader in 384 multiwell plates. Three replicates were performed for each data point. Data were reported as normalized percentages of residual activity. The potency (IC_50_) was calculated from the value of the dose response plot corresponding at a fractional activity of 50%. The IC_50_ values were determined using a non linear fit of experimental data in according to equation:


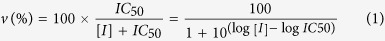


The midpoint of this function occurs at a fractional activity value of 50%, corresponding to half inhibition of the target enzyme. The IC_50_ values and their standard errors were deduced from the fitting. To evaluate if copper is a reversible PI, we measured the ChT-L activity of hCP incubated with Cu(II) (2.20 μM) before and after addition of an equimolar amount of EDTA. The results did not evidence any recovery of the ChT-L activity after EDTA addition thus suggesting that Cu(II) ions may be considered as a mainly irreversible proteasome inhibitors.

### Steady-state fluorescence emission measurements

Fluorescence emission spectra of purified 20S proteasomes (1.5 μM) in a MOPS buffer 10 mM, 0.018% SDS, pH 7.6 were recorded at 37 °C over time prior to and following addition of Cu(II) (100 μM). All spectra were corrected by subtraction of the background buffer spectrum obtained under identical experimental conditions. The final fluorescence spectra represent averages of at least three measurements. Bandwidths of 2 nm for both excitation and emission were used. Fluorescence spectra were recorded on a Horiba Fluoromax-4 spectrofluorimeter from 300 to 400 nm in 1-nm steps, at an excitation wavelength λ_exc_ = 295 nm. Deconvolution of fluorescence spectra into the log-normal components was performed according to the algorithms integrated into the web-based toolkit PFAST (http://pfast.phys.edu/)^43^. The emission spectra were corrected for the instrumental spectral sensitivity by using an aqueous solution of L-tryptophan (120 μM) as a standard.

### Reactive Oxygen Species assay

Reactive oxygen species (ROS) were detected using the ROS-sensitive probe 5–6 carboxyfluorescein^44^. The measurements were assayed according to cell-free conditions used for proteasomal activity. The decrease of fluorescence intensity of 5–6 carboxyfluorescein (1 μM) at 515 nm (λ_ex_ = 493 nm) due to ROS generation was measured at 37 °C using a Horiba Fluoromax-4 spectrofluorimeter. The excitation and emission band pass was set at 2.0 nm each. Initial fluorescence intensities were normalized.

### Cell cultures

HeLa cells (human cervix carcinoma), were cultured in Dulbecco’s MEM medium (Life Technologies) supplemented with 10% of fetal calf serum (FCS, Life Technologies), glutamax (Invitrogen), 100 U/mL penicillin, 100 g/mL streptomycin. Cells were routinely grown as stocks in 75 cm^2^ flasks in a humidified atmosphere (95% air, 5% CO_2_) at 37 °C.

### Proteasome activity assays in intact cells

The ChT-L proteolytic activity of the proteasome in intact HeLa cells were determined using the Proteasome-Glo™ Chymotrypsin-Like Cell-Based Assay (Promega) according to manufacturer's instructions. Briefly, 800 cells/well were grown in 384 sterile multiwells (optical plate and white walls) in 50 μl of DMEM + FBS10% + PenStrep 1%. After 4 h of cell seeding, Cu(II) was added in cell medium in a concentration range 10–80 μM for 24 h. Then the medium was removed and replaced with FCS free medium. To determine whether or not the residual proteolytic measured was dependent on to proteasome rather than other proteases, we performed a control experiment with epoxomicin, a specific proteasome inhibitor. Epoxomicin (5 μM) was added to the cells 60 min before the proteasome activity assay. The substrate for the β5 ChT-L activity (Suc-LLVY-aminoluciferin) was dissolved in Proteasome-Glo™ Cell-Based Reagent and added to intact cells. After 2 min of shaking for permeabilization and further 10 min of incubation at 25 °C, luminescence was measured with a Varioskan Flash (Thermo) micro plate reader. Proteasome activity in intact cells was also measured by using the internally quenched fluorogenic substrate H_2_N-K-K(Dabcyl)-K-K-L-L-V-Y-G-E(Edans)-G-R-K-K-R-R-Q-R-R-R- (TED) as described elsewhere^36^. The peptide TED was assembled using the microwave-assisted solid phase peptide synthesis strategy on a Liberty Peptide Synthesizer briefly described in the supporting information. The peptide purity was determined by MALDI-TOF experiments carried out with a Bruker Reflex III time-of-flight (TOF) mass spectrometer operating in the reflection mode, and the matrix used was α-cyano 4-hydroxycinnamic. HPLC chromatograms and MALDI-TOF spectra of purified peptides are reported in the supplementary information (see Figures S5, S6 and S7 of Supplementary Information). TED internalization and cleavage in live HeLa cells was assayed by fluorescence microscopy (see Figure S8 of Supplementary Information).

### Western blot analysis

After 24 h of copper(II) treatment (20–80 μM), HeLa cells were washed with cold PBS, and lysed with 100 μL of RIPA buffer for 5 minutes at 4 °C, sonicated and centrifuged for 10 minutes at 4 °C (1400 rpm). The protein content of lysates is quantified using the BCA method, (protein assay kit, BioRad). Western blotting analysis for polyUb chains was performed on total protein extracts (50 g). Samples were loaded onto 4–12% bis-Tris gel, (Invitrogen). After separation, proteins were transferred onto a nitrocellulose membrane. Membranes were blotted at 4 °C O/N with the following primary antibodies: rabbit monoclonal anti-polyubiquitin (linkage specific K48) (1:2500, Abcam), and mouse anti-β-actin (1:1000, Sigma Aldrich). Secondary goat anti-rabbit labeled with IRDye 680 (1:25.000 Li-COR Biosciences) and goat anti-mouse labeled with IRDye 800 (1:25.000 Li-COR Biosciences) were used at RT for 45 min. Hybridization signals were detected with the Odyssey Infrared Imaging System (LI-COR Biosciences).

### Native Gel Electrophoresis

Proteasome-enriched fractions were subjected to non denaturing native gel electrophoresis, as described elsewhere^45^. Briefly, 100 μg of fractionated Hela cells lysates, pretreated for 24 h with 0, 20 or 80 μM Cu(II), were run on a 3–12% non-denaturing, gradient polyacrylamide gel (Invitrogen, Carlsbad, CA) according to a protocol reported elsewhere^46^. hCP (2 μM) treated for 2 h with 0 μM, 20 μM and 80 μM of Cu (II) was used as reference control. Peptidolytic activity of proteasome-enriched fractions from cell lysates was evaluated after incubating the gels in a Suc-LLVY-AMC substrate dissolved in 50 mM Tris pH 8.0, 5 mM MgCl_2_, 1 mM DTT, 1 mM ATP, at 37 °C for 15 min. Proteasome bands were identified by the release of highly fluorescent free AMC which allowed us to identify the proteasome holo and subcomplexes. Images of the gels were acquired by Chemidoc (Biorad), and fluorescent bands were quantified by using the Image J software. Native gel of purified 20S proteasome (0.5 μg) were revealed by Blue Coomassie.

### Flow cytometry

HeLa cells plated into 12-well plates (1.5 × 10^5 ^cells/well), were exposed to increasing Cu(II) amounts (20–100 μM) for 24 h before being stained with Pheen Green following instructions provided by the manufacturer. After washing, cells were harvested and analyzed on a CyFlow ML flow cytometer (Partec), reading the fluorescence emission in FL1 log mode (ex 495 nm/em 535 nm). Only viable cells detected by reading the scattering indicated as FSC and SSC, were considered for our analysis. Roughly 20.000 cells per sample were analyzed using a CyFlow ML flow cytometer (Partec) system equipped with three laser sources and 10 optical parameters with dedicated filter setting and a high numerical aperture microscope objective (50 NA 0.82) for the detection of different scatter and fluorescence signals. Cells were excited by an air-cooled argon 488 nm laser, then the Phen Green signal was read on FL1 detector. Data obtained were acquired, gated, compensated by using the FCS Express 4 software (DeNovo). Reported data are representative for three sets of independent experiments performed in triplicate and based on 20.000 events for each group. The chi-square test was used for statistical analysis. A P value below 0.001 was considered as significant.

## Additional Information

**How to cite this article**: Santoro, A. M. *et al*. Copper(II) ions affect the gating dynamics of the 20S proteasome: a molecular and *in cell* study. *Sci. Rep.* **6**, 33444; doi: 10.1038/srep33444 (2016).

## Supplementary Material

Supplementary Information

## Figures and Tables

**Table 1:** Data fitting (see Fig. 1A) relative to the evaluation of the IC_50_ values of Cu(II) and Zn(II) ions for ChT-L, T-L and C-L peptidase activity of the CP.Curve fitting was performed by using Equation 1.

	ChT-L		T-L		C-L	
	Cu(II)	Zn(II)	Cu(II)	Zn(II)	Cu(II)	Zn(II)
IC_50_ (μM)	1.19	5.99	1.45	4.16	1.01	5.84
95% Confidence Intervals	0.67 to 2.09	2.98 to 12.30	0.98 to 2.14	1.65 to 10.53	0.66 to 1.56	4.94 to 6.90
R^2^	0.91	0.91	0.96	0.81	0.95	0.99

Curve fitting was performed by using Equation 1.
