# Supplementary material for: Copper(II) ions affect the gating dynamics of the 20S proteasome: a molecular and in cell study
Source: Sci Rep. 2016 Sep 16;6:33444. doi: 10.1038/srep33444 (PMC5025780; doi:10.1038/srep33444)
Supplement: Supplementary Information [file srep33444-s1.pdf]

Copper(II) ions affect the gating dynamics of the 20S proteasome: a molecular and *in cell* study.

Anna Maria Santoro[1], Irene Monaco[2], Francesco Attanasio[1], Valeria Lanza[1], Giuseppe Pappalardo[1], Marianna Flora Tomasello,[1] Alessandra Cunsolo[2], Enrico Rizzarelli[1,2], Ada De Luigi[3], Mario Salmona[3] and Danilo Milardi*[1]

[1]*Istituto di Biostrutture e Bioimmagini - CNR Sede di Catania, Via P. Gaifami, 9- 95126 Catania, Italy.*

[2]*Fondazione RiMed, Via Bandiera 11, 90133, Palermo, Italy.*

[3]*Dipartimento di Scienze Chimiche, Università di Catania, Viale Andrea Doria 6, 95125 Catania, Italy.*

[4]*IRCCS-Istituto di Ricerche Farmacologiche "Mario Negri", Via Giuseppe La Masa 19, 20156, Milano, Italy.*

Supplementary information

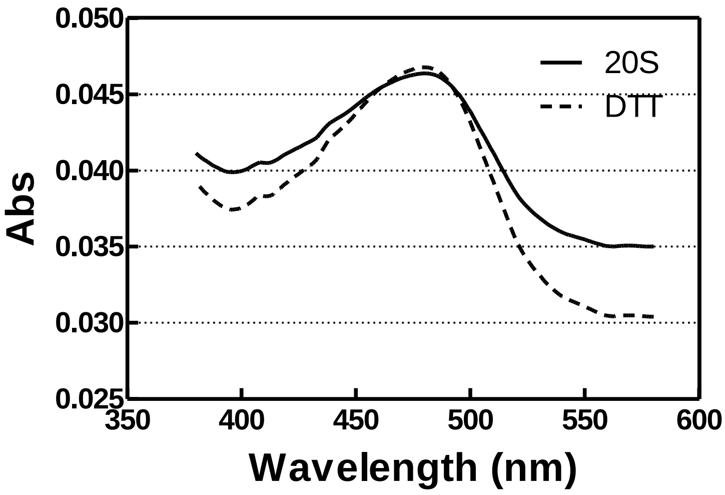

Figure S1. UV bands monitored for a 10 μM Cu(II) solution incubated with 100 μM BCS with (solid line) or without 20S (dashed line). Isolated 20S proteasomes (35 ng/ml) were dissolved in a 10 mM MOPS buffer pH 7.6 containing 100 μM BCS, 1.5 μM Cu(II) and 29 μM DTT. UV spectroscopic measurements were carried out by a UV Jasco J-670 spectrophotometer. UV spectra were recorded in a 350 – 600 nm range with a 1nm step. Optical path was 1 cm.

Due to the presence of oxygen in the test tubes, Cu(II) ions may also catalyze the oxidation of cysteinyl thiols to produce S-S disulfide linkages with a resulting reduction of Cu(II) to Cu(I)[1] and formation of Cu(I) complexes. Hereby Cu(II) may induce the generation of reactive oxygen species (ROS) and convert the Cys into cystine. In a previous report the generation of Cu(I) in Cu(II)-loaded CP samples was investigated by using bathocuproinedisulfonic acid sodium salt (BCS) according to a previously described method. (see Linder, et al Am. J. Clin. Nutr. 1998, 67, 965S; Xiao, et al. Int. J. Oncol. 2010, 37, 81). Indeed, it is known that Cu(I)-BCS complexes exhibit a specific absorption band at 480 nm which may be used to monitor the reduction of Cu(II) to Cu(I).

[1] Rigo, A., Corazza, A., Di Paolo, M.L., Rossetto, M., Ugolini, R. M. & Scarpa, M. Interaction of copper with cysteine: stability of cuprous complexes and catalytic role of cupric ions in anaerobic thiol oxidation, *J.Inorg.Biochem*. 98, 1495 (2004).

Isolated 20S proteasome may promote the reduction of Cu(II) to Cu(I) species thus suggesting that the CP has an intrinsic reducing capacity. However, most of the commercially available 20S proteasome preparations, including those used in the reference study are delivered in a DTT-enriched buffer which, due to its known reducing potential, may interfere with the assay. In order to ascertain if DTT may by itself promote the reduction of Cu(II) to Cu(I) we prepared a CP-free aqueous mimicking the DTT-enriched 20S proteasome solutions used in the UV studies (Fig S5). Our analysis on one hand confirmed the previously obtained results, but also evidenced that the UV band observed at 480 nm may be merely ascribed to the reducing effect of DTT. These data suggest that the hypothesized 20S-induced reduction of Cu(II) to Cu(I) is not confirmed in the experimental conditions here adopted. Furthermore, we have observed that BCS is also able to catalyze the formation of BCS-Cu(I) species even in the absence of any reducing agent. Therefore this assay cannot confirm the existence of Cu(I) species in CP assays.

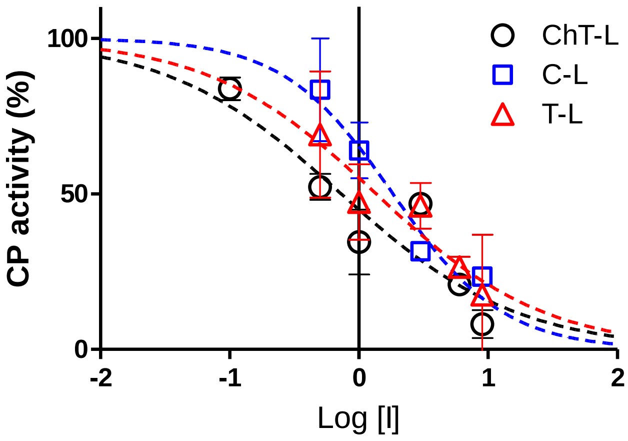

Figure S2. The three CP peptidase activities obtained in the presence of 0.018% SDS as a semilog plot fitted by equation 1. All the other experimental conditions were unmodified respect the results reported in Fig 1A and Table 1 in the main main text. IC_{50} values for the distinct peptidase activities and the related fitting parameters are reported in Table S1.

Table S1 Data fitting (see Figure S2) relative to the evaluation of the IC_{50} values of Cu(II) ions for ChT-L, T-L and C-L peptidase activity of the CP measured in the presence and in the absence of 0.018% SDS. Curve fitting was performed by using equation 1.

**20S peptidase activity**	ChT-L		T-L		C-L	
	**Cu(II)**	**Cu(II) + SDS**	**Cu(II)**	**Cu(II) + SDS**	**Cu(II)**	**Cu(II) + SDS**
**IC_{50} (μM)**	1.19	0.73	1.45	1.35	1.01	1.83
**95% Confidence Intervals**	0.67 to 2.09	0.24 to 2.18	0.98 to 2.14	0.61 to 2.99	0.66 to 1.56	0.83 to 4.02
**R^2**	0.91	0.84	0.96	0.89	0.95	0.9552

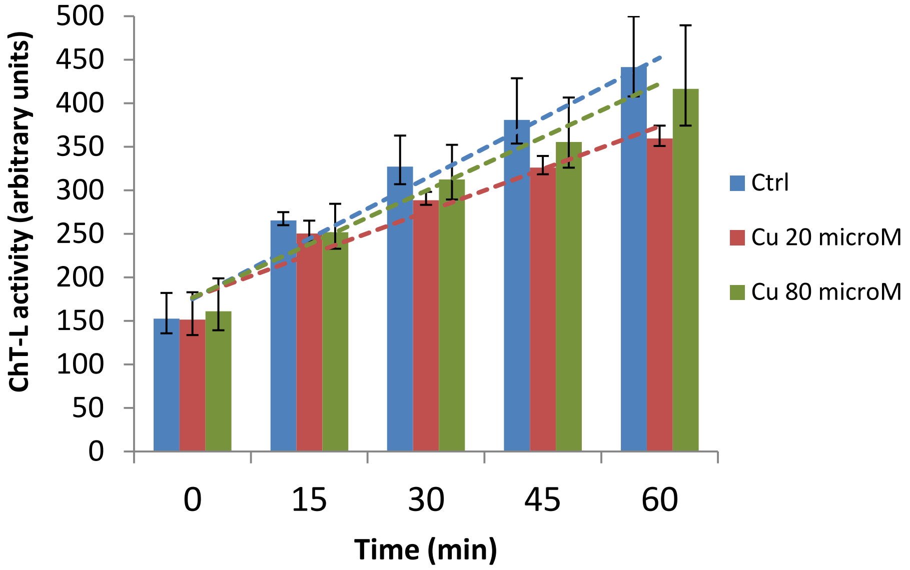

Figure S3. The ChT-L proteasome activity of intact HeLa cells incubated with 20 (red bars) and 80 (green bars) μM Cu(II) was monitored vs time by using the proteasome activity reporter TED.

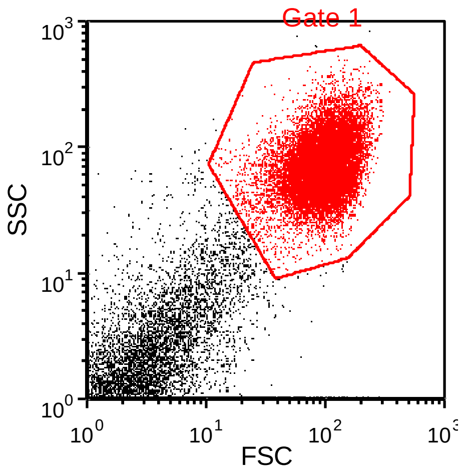

A

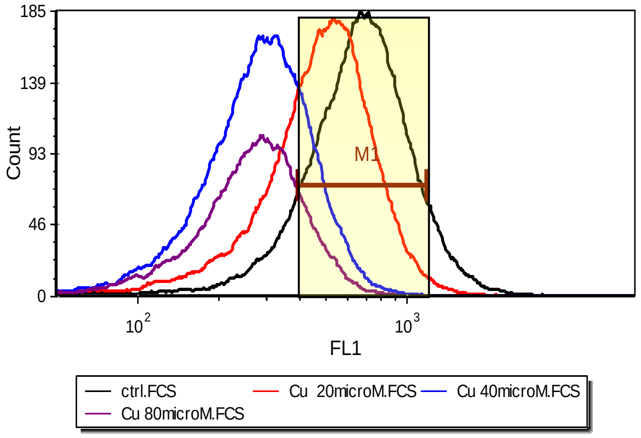

B

Figure S4 A). Representative flow cytometry 2D plots: cells were treated with the indicated Cu (II) concentration for 24h, and stained with Pheen Green. After washing, cells were analysed by Flow cytometry, reading the scattering indicated as FSC and SSC. Only viable cells included in the R1 area were considered for analysis. B). Representative Flow cytometry 1D plots: cells were treated with the indicated Cu (II) concentration for 24h, and stained with Pheen Green. After washing, cells were analysed by Flow cytometry, reading the fluorescence on FL1 channel (λex 495 nm/λem 535 nm). In Cu (II) treated cells the green fluorescent signal drops in a dose-dependent manner.

Synthesis and characterization of the fluorogenic peptide TED.

All Fmoc-amino acids were introduced according to the TBTU/HOBT/DIEA activation method. All synthesis was carried out under a 4-fold excess of amino acid. Removal of Fmoc protection during synthesis was achieved by means of 20% piperidine solution in DMF. The following instrumental conditions were used for each coupling cycle: microwave power 25 Watts, reaction temperature 75 °C, coupling time 300 sec. The instrumental conditions used for the deprotection cycles were: microwave power 25 Watts, reaction temperature 75 °C, deprotection time 180 sec. This peptide was assembled using a Fmoc-Arg(pbf)-TGA resin with attached the first amino acid of the sequence (substitution 0.23 mmol/g). After the completion of the coupling cycles, the peptidyl-resin H_2N-K-K(Dabcyl)-K-K-L-L-V-Y-G-E(Edans)-G-R-K-K-R-R-Q-R-R-R-TGA was treated with a mixture of TFA/TIS/H2O (95/2.5/2.5 v/v) for 1 hour at room temperature to give the peptide without resin. The solution containing the free peptide was filtered off from the resin and concentrated in vacuo at 30 °C. The peptide was precipitated with cold freshly distilled diethyl ether, then filtered and dried under vacuum. The resulting crude peptide was purified by RP-HPLC and characterized by MALDI-TOF MS. Analytical Reversed-Phase High-Performance Liquid Chromatography (RP-HPLC) analyses were performed using a Zorbax Eclipse XDB-C184.6x150 mm (5µm particle size) column at a flow rate of 1 ml/min. While, preparative RP-HPLC was carried out by means of Varian PrepStar 200 model SD-1 chromatography system equipped with a Prostar photodiode array detector on a Vydac C18 250x22 mm (300 Å pore size, 10-15 µm particle size) column at flow rate of 10 mL/min. Detection in both cases was at 222 nm. HPLC eluents were A: 0.1% TFA/water and B: 0.1% TFA/acetonitrile. The peptide sample was analysed using the following gradient elution with solvents A and B: Minutes 0, 5, 15, 20, 30, 35 with % CH_3CN 5, 5, 30, 30, 5, 5, respectively. The molecular weight of peptide TED is 3084 Da. The yield of the synthesis was 60 %. The obtained crude TED peptide was purified by RP-HPLC and characterized by MALDI-TOF MS. Figures S1 and S2 show respectively the chromatograms relatives to the crude and purified peptide.

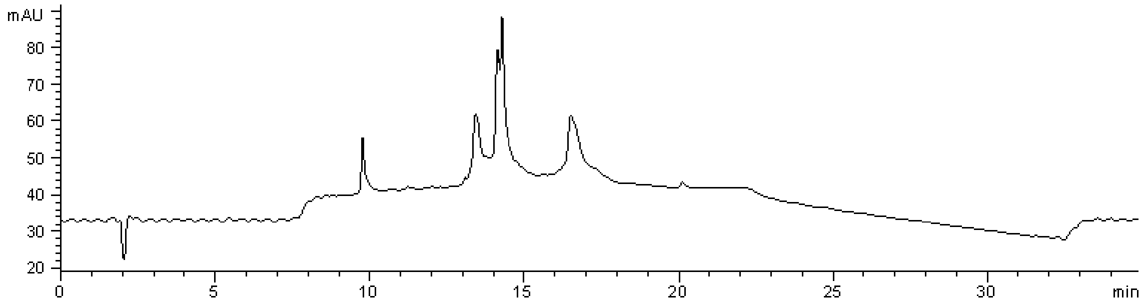

Figure S5. HPLC chromatogram for crude TED.

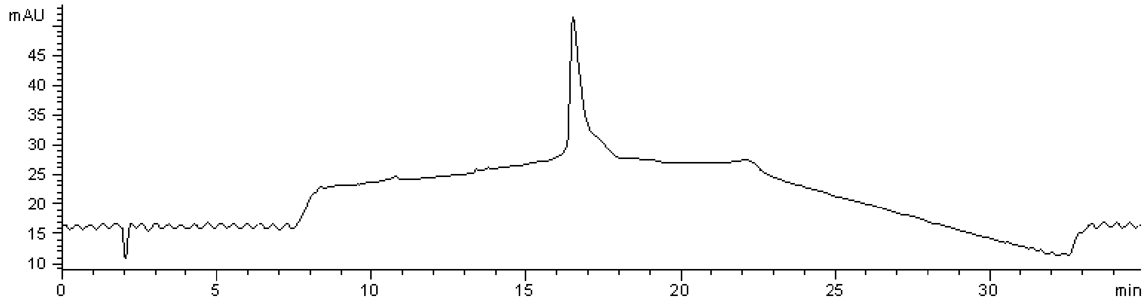

Figure S6. HPLC chromatogram for purified TED.

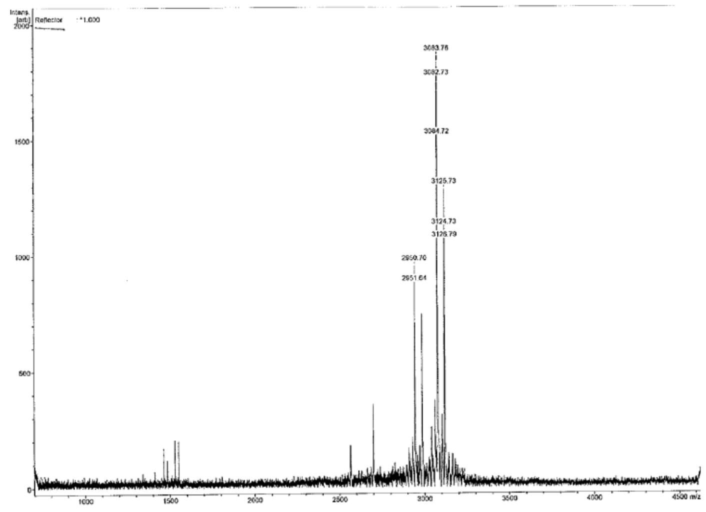

Figure S7. MALDI mass spectra for TED peptide. MALDI spectra that results in a number of peaks with recorded m/z values derived from the peptide TED. Two peaks are present in the spectra: one, more intense, is the peptide TED, the other, with m/z value 2950, show a mass 134 units lower than TED.

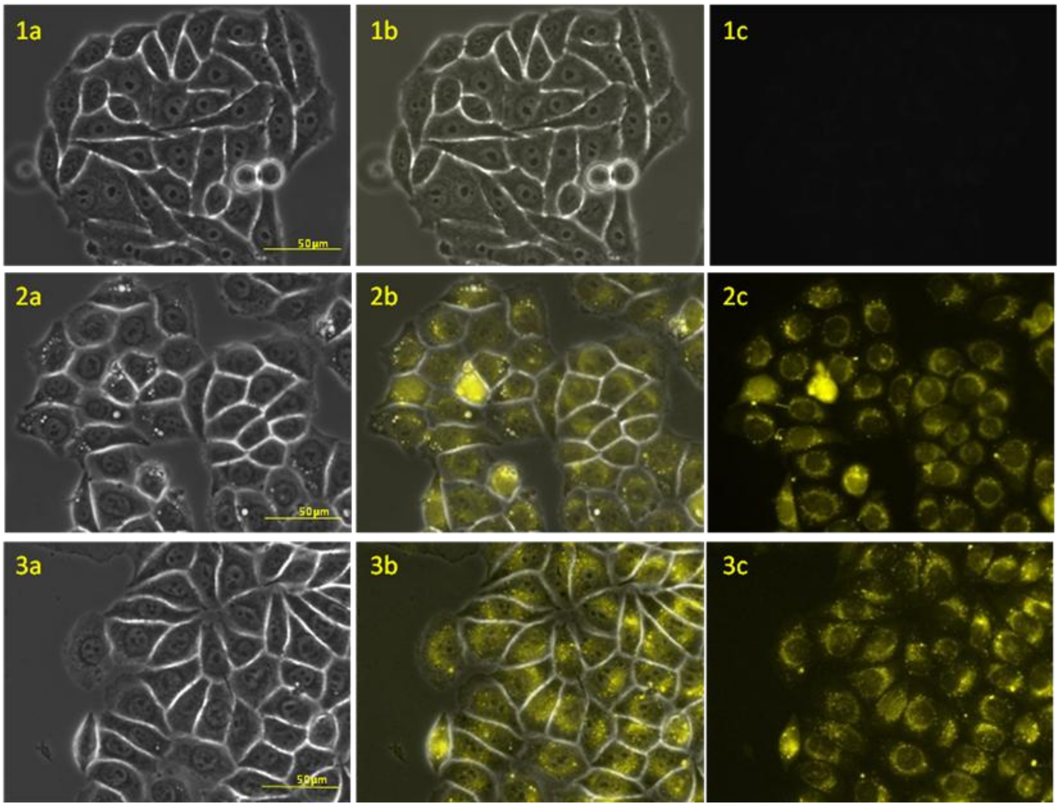

Figure S8. TED internalization and cleavage in HeLa cells, EDANS fluorescence (yellow), 1: HeLa cells untreated (control) , 2: HeLa cells incubated for 30 min with TED 50 µM, 3: HeLa cells incubated for 60 min with TED 50 µM.
